# GLP-1 receptor agonist ameliorates experimental lung fibrosis

**DOI:** 10.1038/s41598-020-74912-1

**Published:** 2020-10-22

**Authors:** Juan Fandiño, Laura Toba, Lucas C. González-Matías, Yolanda Diz-Chaves, Federico Mallo

**Affiliations:** Laboratory of Endocrinology (LabEndo), The Biomedical Research Centre (CINBIO), University of Vigo, Campus Universitario de Vigo (CUVI), 36310 Vigo, Spain

**Keywords:** Animal disease models, Respiration, Respiratory tract diseases, Translational research

## Abstract

Idiopathic pulmonary fibrosis (IPF) is a chronic, progressive, and fatal lung disease. This disease is characterized by an excessive accumulation of extracellular matrix deposition that modify normal lung physiology. Up to date, there are not efficient therapeutic tools to fight IPF. Glucagon-like peptide-1 receptor (GLP-1R) activation plays an essential role in lung functions in normal and in pathological conditions. The aim of the present study was to study the possible beneficial effects of the administration of the GLP-1R agonist, liraglutide, in the pathogenesis of the fibrotic process in an animal model of pulmonary fibrosis induced by bleomycin. We observed that liraglutide decreased mRNA expression of collagen, hydroxyproline and key enzymes for the synthesis of collagen. In addition, GLP-1R activation restored the ACE2 mRNA levels modulating the activities of the RAS components, increased the production of surfactant proteins (SFTPa1, SFTPb, SFTPc) and promoted an improvement in pulmonary and cardiac functionality, including a partial restoration of lung alveolar structure. Liraglutide effects are shown at both the pro-inflammatory and fibrosis phases of the experimental disease. For these reasons, GLP-1 might be regarded as a promising drug for treating pulmonary fibrosis.

## Introduction

Extracellular matrix (ECM) is a non-cellular component composed by proteoglycans and fibrous proteins. Collagen is the most abundant fibrous protein in the interstitial ECM and constitutes up to 30% of the total protein mass of a multicellular animal^1^. Alterations in the tissue deposition of ECM or its components, especially collagen species, promotes structural and functional defects of the organs compiled as fibrosis.

Idiopathic pulmonary fibrosis (IPF) is a chronic, progressive, and finally fatal lung disease of unknown etiology. It consists in alveolar-epithelial cells micro injuries caused by environmental factors that triggers an aberrant cell activation, promotes the migration of mesenchymal cells and the formation of fibroblast and myofibroblast foci^2^. Myofibroblasts are a heterogeneous population of cells that may derived from a large variety of cell types, such as epithelial-mesenchymal transition, circulating fibrocytes and local mesenchymal cells^3^. They are present in physiological and pathological conditions, and they are responsible for the extracellular matrix production^4^. In normal wound healing process, myofibroblasts are later eliminated by apoptosis, however, in the fibrotic state they are resistant to apoptosis and persist in time, promoting an excessive depot of ECM^2^. Myofibroblasts in the fibroblastic foci can induce the formation of large and rigid collagen bundles, which disrupts the basal membrane and comprises tissue integrity^1^ driving to interstitial fibrosis. Up to date, there are not efficient therapeutic tools to fight IPF. In fact, there are just two drugs approved for the treatment of IPF: pirferidone and nintedanib, which only delay the progression rhythm of the disease, with a very limited improvement of symptoms and no increase of survival^5^.

There are different experimental models of pulmonary fibrosis, such as radiation damage, transgenic mice expressing fibrogenic cytokines, and instillation of BLM silica or asbestos^6^. The use of bleomycin (BLM) to produce IPF is one of the most widely extended and accepted models to study this pathology; easily accessible and reproducible^6^. Bleomycin is a chemotherapeutic antibiotic, which causes an inflammatory reaction in the pulmonary alveoli when it is inhaled. This initial reaction switches to a fibrotic response around day 9 after administration^7^. Bleomycin reproduces typical features of IPF.

On the other hand, Glucagon-like peptide 1 (GLP-1) is an insulinotrophic hormone mainly produced by enteroendocrine L-cells of the ileum in response to food intake^8^, but also produced in the brain, especially in the hypothalamus. GLP-1 acts by binding to GLP-1 receptor (GLP-1R), a G-protein-coupled receptor that is widely expressed in many tissues, including a very high expression in lung^9^. GLP-1R is expressed in different areas of lung tissue, such as submucosal glands of the trachea, smooth muscle of pulmonary arteries and in alveolar type II cells (ATII)^9,10^. GLP-1R activation plays an essential role in lung functions in normal and in pathological conditions, as it stimulates in vitro phosphatidyl-choline secretion in ATII cells cultured^11,12^. On in vivo studies, GLP-1R activation during pregnancy, increases Surfactant protein A and B expression in foetus^13^ and the SFTPs production by ATII cells in normal newborns and pups with lung hypoplasia induced by nitrofen^13^; where it does indeed at least as much as dexamethasone. Additionally, the GLP-1R agonist liraglutide (LIR) restores the altered alveoli structure of hypoplasic lung. Moreover, GLP-1R activation restores surfactant protein levels in a type 1 diabetes animal model^14^.

Very remarkable, LIR increases the expression of angiotensin I converting Enzyme 2 mRNA (*Ace2*) in lungs, thus activating the branch axis ACE2/Ang (1–7)/MAS1 of the Renin-Angiotensin System (RAS), which improves pulmonary vascular function. In fact, LIR restores the imbalance of the renin-angiotensin system improving pulmonary vascular function and then reverting the right ventricular hypertrophy, just in a week of treatment in diabetic rats^14^. This effect in the ACE2/Ang (1–7)/MAS1 axis was also shown in a model of intrauterine growth retardation induced by mother-food restriction, suggesting that GLP-1 has a very important role as regulator of RAS^15^. GLP-1 also attenuates lipopolysaccharide acute lung-injury in mice^16^ and reverses pulmonary arterial hypertension induced by monocrotaline in rats^17^.

Previous studies have demonstrated that GLP-1R activation might play a role in tissue fibrosis in different organs. In an experimental model of renal fibrosis, GLP-1R activation prevents epithelial-mesenchymal transition and ECM deposition by inhibition of TGF-β1/Smad3 and ERK1/2 signalling pathways^18^. In addition, dipeptidyl peptidase-4 inhibition, an enzyme that degrades endogenous GLP-1, and GLP-1R activation can reverse cardiac fibrosis induced by Angiotensin II by restoring angiotensin 2 type 2 receptor (AGTR2)/ACE2 imbalance^19^.

Taken all together these potent effects of GLP-1 in lung architecture, function and pathophysiology, the GLP-1R agonist family of peptides became a promising candidate to be tested as potential therapeutic agents in pulmonary fibrosis, once shown they are able to modulate many of the mechanisms underlying the pathophysiology of this disease. In this work, we addressed the effects of GLP-1 in lung inflammation, collagen deposition, RAS activity and histological changes in a recognized experimental model of lung fibrosis induced by bleomycin.

## Results

### Effects of bleomycin instillation and liraglutide treatment over body weight variation

Rats instilled with BLM lost weight until day 5 and gained it afterwards (Supplementary Fig S1a). LIR did not promote differences in body weight gain in control animals at given doses. However, LIR administration increased body weight gain in BLM-treated rats, with significant differences from day 15 to day 21 (Supplementary Fig. S1a). Moreover, the slope of the body weight variations was also significantly higher in the BLM/LIR group (Supplementary Fig. S1b) respect to all others, reflecting a catch-up in body weight.

### Bleomycin upregulates mRNA expression of pro-fibrotic markers and *Tgfb1* levels; and liraglutide treatment prevents this effect

**Figure 1:**
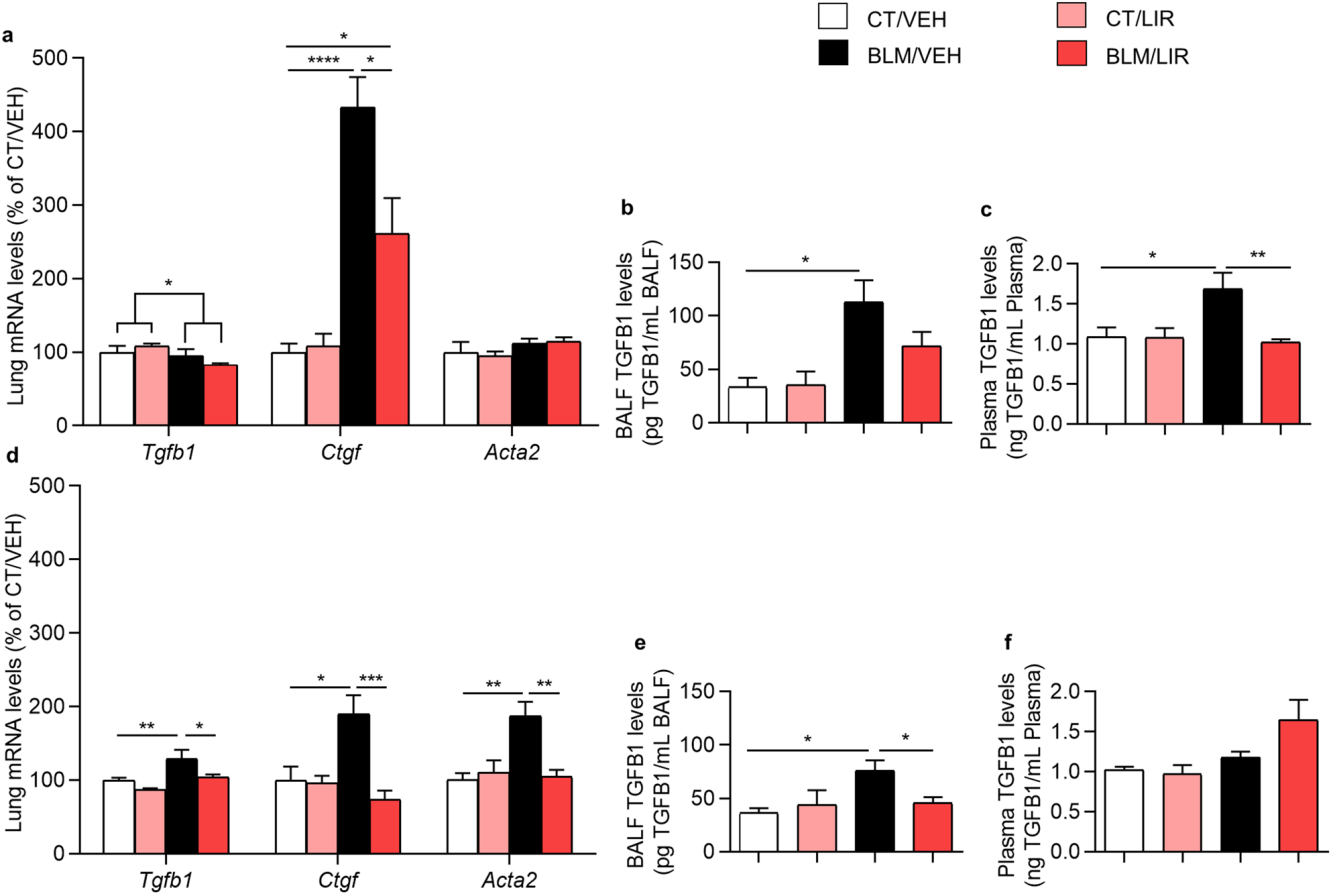
Lung mRNA expression of fibrosis markers normalized to CT/VEH group. Bars represent mean, and error bars SEM. Two-way ANOVA following Bonferroni’s multiple comparison test unless otherwise specified. *p ≤ 0.05, **p ≤ 0.01, ***p ≤ 0.001, ****p ≤ 0.0001. (**a**) Day 7 mRNA levels of transforming growth factor beta-1 (*Tgfb1*; significance represents two-way ANOVA result), connective tissue growth factor (*Ctgf*) and actin, alpha 2, smooth muscle (*Acta2*) normalized to CT/VEH group. n = 6–8 per group. (**b**) Day 7 bronchoalveolar lavage (BAL) TGFB1 levels. Values represent pg of TGFB1 per mL of BAL. n = 6–12 per group. (**c**) Day 7 plasma TGFB1 levels. Values represent ng of TGFB1 per mL of plasma. n = 4–11 per group. (**d**) Day 21 mRNA levels of *Tgfb1*, *Ctgf* and *Acta2* normalized to CT/VEH group. n = 6–8 per group. (**e**) Day 21 BAL TGFB1 levels. Values represent pg of TGFB1 per mL of BAL. n = 4–10 per group. (**f**) Day 21 plasma TGFB1 levels. Values represent ng of TGFB1 per mL of plasma. n = 4–10 per group.

Bleomycin instillation decreased mRNA expression of transforming growth factor, beta 1 (*Tgfb1)* in the inflammatory phase (Day 7, *p* = 0.0338) and it markedly increased the mRNA levels of connective tissue growth factor (*Ctgf*) (4.33-fold increase vs. CT/VEH, *p* ≤ 0.0001, Fig. 1a). The expression levels of the other marker studied, actin alpha 2, smooth muscle (*Acta2)* remained unchanged. LIR treatment significantly reduced the increased levels of *Ctgf* (1.4-fold decrease BLM/LIR vs. BLM/VEH groups, *p* = 0.0121, Fig. 1a).

Bleomycin instillation significantly increased day 7 BAL and plasma TGFB1 levels respect to CT/VEH group (BAL: 112.97 ± 20.32 pg/mL vs. 33.49 ± 8.6 pg/mL, *p* = 0.0156, Fig. 1b. Plasma: 1.69 ± 0.2 ng/mL vs. 1.09 ± 0.12 ng/mL, *p* = 0.0304, Fig. 1c). LIR treatment partially reduced BAL TGFB1 levels (71.77 ± 13.1 pg/mL, NS; Fig. 1b) and completely normalize plasma TGFB1 levels (1.02 ± 0.04 ng/mL, *p* = 0.002, Fig. 1c), at that experimental time.

The mRNA levels of *Tgfb1* (1.38-fold increase, *p* = 0.0088), *Ctgf* (1.89-fold increase, *p* = 0.017) and *Acta2* (1.87-fold increase, *p* = 0.0025) were markedly increased in the BLM-administered animals respect to CT/VEH in day 21 (fibrotic phase, Fig. 1d). And LIR treatment completely restored the levels of the three markers with respective decreases of *Tgfb1* (1.20-fold decrease BLM/LIR vs. BLM/VEH, *p* = 0.0232), *Ctgf* (1.58-fold decrease BLM/LIR vs. BLM/VEH, *p* = 0.0006) and *Acta2* (1.44-fold decrease BLM/LIR vs. BLM/VEH, *p* = 0.002, Fig. 1d). At day 21, BAL TGFB1 levels were also markedly increased in BLM/VEH respect to CT/VEH group (75.73 ± 9.84 pg/mL vs. 36.44 ± 4.33 pg/mL, *p* = 0.0149, Fig. 1e) whereas no variation in plasma TGFB1 levels was found (Fig. 1f). LIR treatment completely normalizes TGFB1 levels (45.58 ± 5.66 pg/mL vs. 75.73 ± 9.84 pg/mL, *p* = 0.0499, Fig. 1e).

### Liraglutide treatment in extracellular matrix synthesis

**Figure 2:**
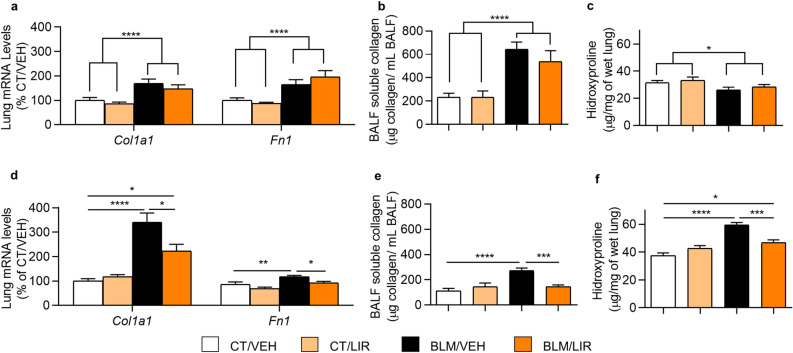
Extracellular matrix elements. Bars represent mean, and error bars SEM. Two-way ANOVA following Bonferroni´s multiple comparison test unless otherwise specified. *p ≤ 0.05, **p ≤ 0.01, ***p ≤ 0.001, ****p ≤ 0.0001. (**a**) Day 7 mRNA levels of collagen type I alpha 1 chain (*Col1a1*) and fibronectin-1 (*Fn1*) normalized to CT/VEH group. n = 6 per group. Significance represents two-way ANOVA result. (**b**) Day 7 total bronchoalveolar lavage (BAL) soluble collagen. Values represent µg of soluble collagen per mL of BAL. n = 7–10 per group. Significance represents two-way ANOVA result. (**c**) Day 7 hydroxyproline (OH-Pro) levels in lung. Values represent µg of OH-Pro per mg of wet lung tissue. n = 6–8 per group. Significance represents two-way ANOVA result. (**d**) Day 21 mRNA levels of *Col1a1* and *Fn1* normalized to CT/VEH group. n = 6–8 per group. (**e**) Day 21 total BAL soluble collagen. Values represent µg of soluble collagen per mL of BAL. n = 7–10 per group. (**f**) Day 21 lung OH-Pro levels in lung. Values represent µg of OH-Pro per mg of wet lung tissue. n = 6–8 per group**.**

In the inflammatory phase (day 7), BLM-administered rats showed increased levels of the mRNA of collagen type I alpha 1 chain (*Col1a1*, *p* = 0.0001) and Fibronectin 1 (*Fn1*, *p* ≤ 0.0001, Fig. 2a). In this phase the LIR treatment did not modify the altered mRNA levels of *Col1a1* neither *Fn1* in BLM-administered rats (Fig. 2a). Bronchoalveolar lavage (BAL) soluble collagen levels were greatly increased after bleomycin administration (*p* < 0.0001, Fig. 2b) and LIR treatment was ineffective to restore them at that time (Fig. 2b). Total hydroxyproline (OH-Pro) levels were paradoxically decreased in day 7 after BLM instillation (*p* = 0.0178, Fig. 2c).

However, in the fibrotic phase (day 21), BLM-instilled animals showed increased mRNA levels of the two major components of the ECM (*Col1a1*: 3.4-fold increase vs CT/VEH, *p* ≤ 0.0001; and *Fn1*: 1.18-fold increase, *p* = 0.006, Fig. 2d). LIR reduced their mRNA expression (*Col1a1* 1.34-fold decrease vs. BLM/VEH, *p* = 0.0258; *Fn1* 1.22-fold decrease BLM/LIR vs. BLM/VEH, *p* = 0.0131, Fig. 2d). BLM-instilled animals had increased levels of soluble collagen in BAL (271.42 ± 22.01 vs. 113.08 ± 20.22 μg collagen/ mL BAL, *p* ≤ 0.0001, Fig. 2e). LIR treatment completely restored them (BLM/LIR: 146.27 ± 13.27 vs. BLM/VEH: 271.42 ± 22.01 μg collagen/mL BAL, *p* = 0.0002, Fig. 2e). BLM instillation increased lung OH-Pro levels (BLM/VEH: 59.4 ± 1.9 vs VEH/VEH: 37.6 ± 1.8 μg OH-Pro/mg of wet lung tissue, *p* ≤ 0.0001, Fig. 2f). Liraglutide treatment significantly reduced total lung OH-Pro levels (BLM/LIR: 46.8 ± 2.0 vs BLM/VEH: 59.4 ± 1.9 μg OH-Pro/ mg of wet lung tissue, *p* = 0.0003, Fig. 2f).

### Liraglutide treatment reduces the expression and activity of key enzymes in the synthesis of collagen local precursors

**Figure 3:**
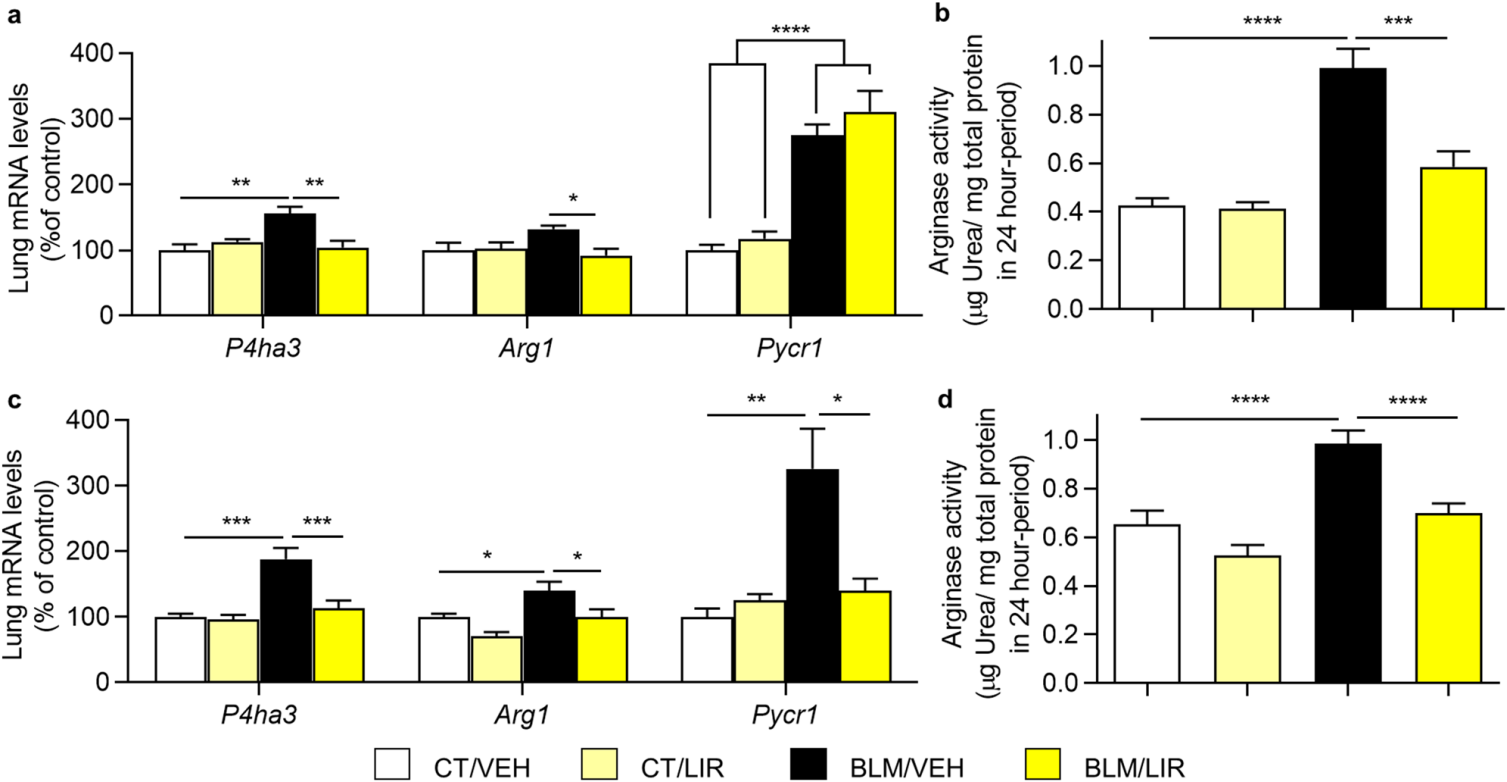
Key enzymes of pathways for synthesis of collagen local precursors. Bars represent mean, and error bars SEM. Two-way ANOVA following Bonferroni´s multiple comparison test unless otherwise specified. *p ≤ 0.05, **p ≤ 0.01, ***p ≤ 0.001, ****p ≤ 0.0001. (**a**) Day 7 mRNA levels of Prolyl 4-hydroxylase subunit alpha 3 (*P4ha3*), arginase 1 (*Arg1*) and pyrroline-5-carboxylate reductase 1 (*Pycr1*) normalized to CT/VEH group. n = 6–8 per group. In *Pycr1* significance represents two-way ANOVA result. (**b**) Day 7 Arginase enzymatic activity in lung tissue homogenate. Values represent μg of urea generated per mg of total protein in tissue homogenate in a 24-h period. n = 6–10 per group. (**c**) Day 21 mRNA levels of *P4ha3*, *Arg1* and *Pycr1* normalized to CT/VEH group. n = 6–7 per group. (**d**) Day 21 Arginase enzymatic activity in lung tissue homogenate. Values represent μg of urea generated per mg of total protein in tissue homogenate in a 24-h period. n = 7–12 per group.

Bleomycin instillation increases day 7 prolyl 4-hydroxylase subunit alpha 3 (*P4ha3*) and pyrroline-5-carboxylate reductase 1 (*Pycr1*) mRNA expression (*P4ha3*, 1.65-fold increase vs. CT/VEH group, *p* = 0.0021; *Pycr1*, *p* ≤ 0.0001, Fig. 3a) but not modify arginase 1 (*Arg1*) mRNA expression levels. LIR treatment normalized the expression of *P4ha3* (1.47-fold decrease vs BLM/VEH, *p* = 0.0021), also reduced *Arg1* (1.31-fold decrease vs. BLM/VEH, *p* = 0.0221), but did not modify *Pycr1* mRNA expression (Fig. 3a). Arginase activity was also markedly increased in BLM-instilled animals (0.96 ± 0.05 vs. 0.44 ± 0.02 µg of urea produced in 24 h/ mg of protein, *p* ≤ 0.0001, Fig. 3b), and liraglutide administration completely restored arginase activity (0.59 ± 0.04 µg of urea produced in 24 h/mg of protein vs. BLM/VEH, *p* = 0.0002, Fig. 3b).

In day 21, BLM-instilled animals have a higher mRNA expression of these three enzymes (*P4ha3* 1.88-fold increase vs. CT/VEH, *p* = 0.0002; *Arg1* 1.4-fold increase vs. CT/VEH group, *p* = 0.0232; *Pycr1* 3.26-fold increase vs. CT/VEH, *p* = 0.0037, Fig. 3c). LIR treatment completely restored their expression levels (*P4ha3* 1.75-fold decrease vs BLM/VEH, *p* = 0.0008; *Arg1* 1.40-fold decrease vs. BLM/VEH, *p* = 0.0177; *Pycr1* 1.86-fold decrease vs. BLM VEH, *p* = 0.0109, Fig. 3c). Arginase activity remains higher in BLM-administered animals (0.94 ± 0.04 vs. 0.65 ± 0.04 µg of urea produced in 24 h/mg of protein, *p* ≤ 0.0001, Fig. 3d). LIR treatment also normalized arginase activity (0.7 ± 0.03 µg of urea produced in 24 h/mg of protein vs. BLM/VEH, *p* ≤ 0.0001, Fig. 3d).

### Liraglutide treatment decreases day 21 collagen deposition and severity of fibrosis

**Figure 4:**
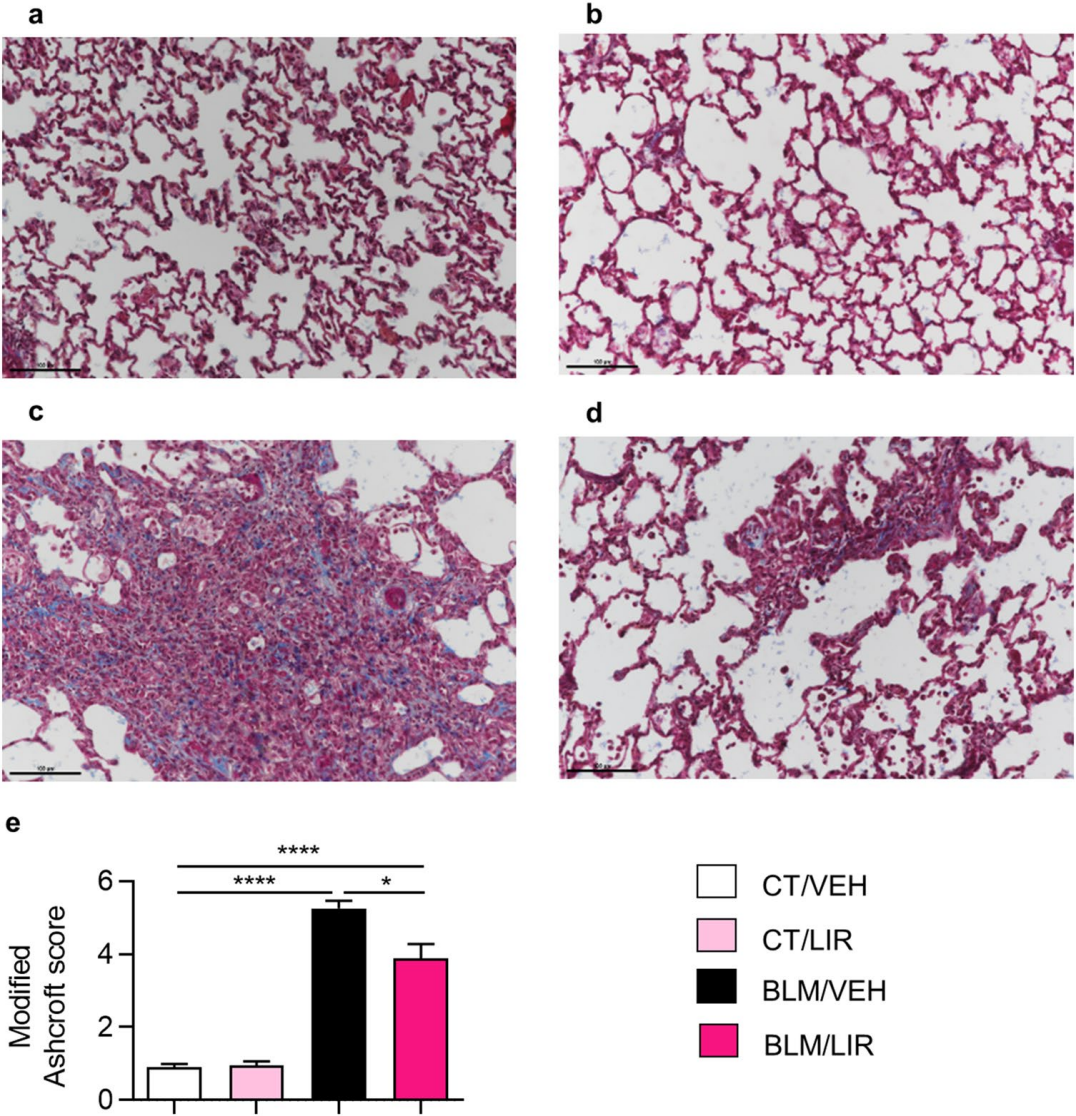
Masson´s Trichrome staining representative microphotographs (200X) and severity of fibrosis. Two-way ANOVA following Bonferroni’s multiple comparison test. *p ≤ 0.05, ****p ≤ 0.0001. (**a**) CT/VEH group. (**b**) CT/LIR group. (**c**) BLM/VEH group. (**d**) BLM/LIR group. (**e**) Severity of fibrosis evaluated by modified Ashcroft score. Bars represent mean, and error bars SEM. n = 3–6 per group**.**

VEH-instilled controls showed the expected lung tissue morphology and no interstitial collagen deposits (Fig. 4a,b). BLM instillation completely altered normal lung architecture a strongly increased collagen fibres deposit in the interstitial space; losing alveoli sacs with appearance of condensed tissue (see the intense blue in the interstitial space, Fig. 4c). BLM instillation strongly increases the severity of fibrosis revealed by the modified Ashcroft score (5.25 ± 0.22 vs. 0.91 ± 0.06, *p* ≤ 0.0001, Fig. 4e). Liraglutide treatment in BLM instilled animals markedly preserved the lung histology and ameliorated alveolar architecture with fewer collagen fibres deposit in interstitial space (Fig. 4d). Accordingly, liraglutide treatment significantly reduced the degree of fibrosis (modified Ashcroft score. BLM/LIR: 3.89 ± 0.32 vs. BLM/VEH: 5.25 ± 0.22, *p* = 0.011, Fig. 4e).

### Liraglutide treatment completely restores day 21 mRNA expression of surfactant-associated proteins and the transcription factor *Nkx2-1*

**Figure 5:**
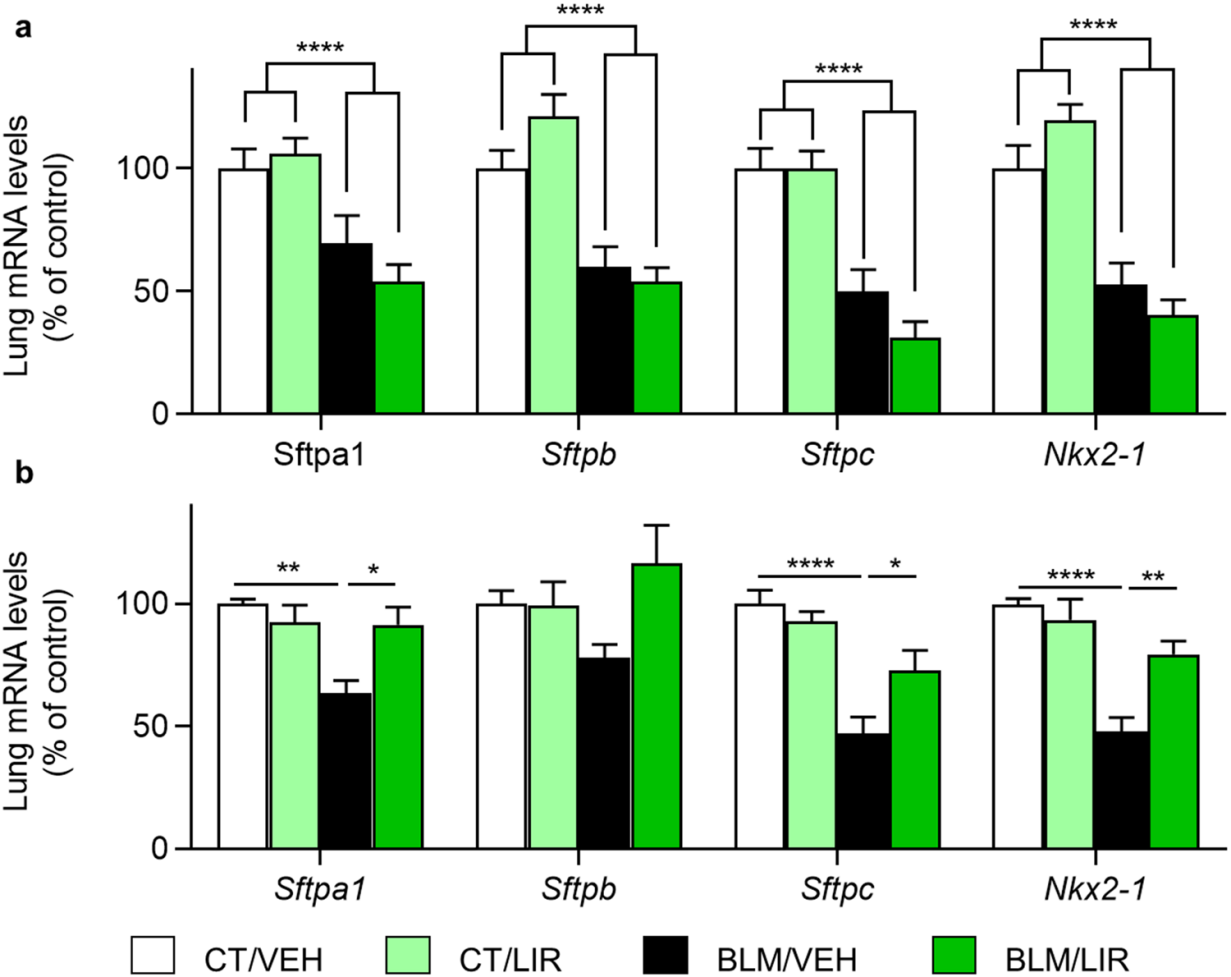
mRNA expression of surfactant proteins normalized to CT/VEH group. Bars represent mean, and error bars SEM. Two-way ANOVA following Bonferroni´s multiple comparison test unless otherwise specified. *p ≤ 0.05, **p ≤ 0.01, ****p ≤ 0.0001. (**a**) Day 7 mRNA levels of surfactant protein A1 (*Sftpa1*), surfactant protein B (*Sftpb*), surfactant protein C (*Sftpc*) and NK2 homeobox 1 (*Nkx2-1*). n = 6–8 per group. Significancy represents two-way ANOVA result. (**b**) Day 21 mRNA levels of *Sftpa1*, *Sftpb*, *Sftpc* and *Nkx2-1*. n = 6–8 per group.

On day 7, BLM instillation reduces mRNA expression of *Sftpa1* (*p* ≤ 0.0001), *Sftpb* (*p* ≤ 0.0001), *Sftpc* (*p* ≤ 0.0001) and *Nkx2-1* (*p* ≤ 0.0001, Fig. 5a). LIR treatment was not effective at this phase to increase the mRNA levels of none of SFTPs nor *Nkx2-1* (Fig. 5a).

On day 21, BLM-instilled animals have a decreased mRNA expression levels of *Sftpa1*, *Sftpc* and *Nkx2-1* genes, in comparison to CT/VEH group (*Sftpa1* 1.36-fold decrease, *p* = 0.0014; *Sftpc* 1.53-fold decrease, *p* ≤ 0.0001; *Nkx2-1* 1.52-fold decrease, *p* ≤ 0.0001, Fig. 5b). At this phase LIR completely restored the mRNA expression levels of SFTPs in BLM-administered rats (*Sftpa1*: 1.44-fold increase, *p* = 0.0161; *Sftpc*: 1.54-fold increase, *p* = 0.0498 *Nkx2-1* 1.67-fold increase, *p* = 0.0018, Fig. 5b). Bleomycin did not significantly reduce the mRNA expression of *Sftpb*, however, liraglutide-treated animals showed a substantial increment of *Sftpb* mRNA expression levels vs. BLM/VEH (1.49-fold increase, NS).

### Effect of liraglutide treatment in the components of the angiotensin system mRNA

**Figure 6:**
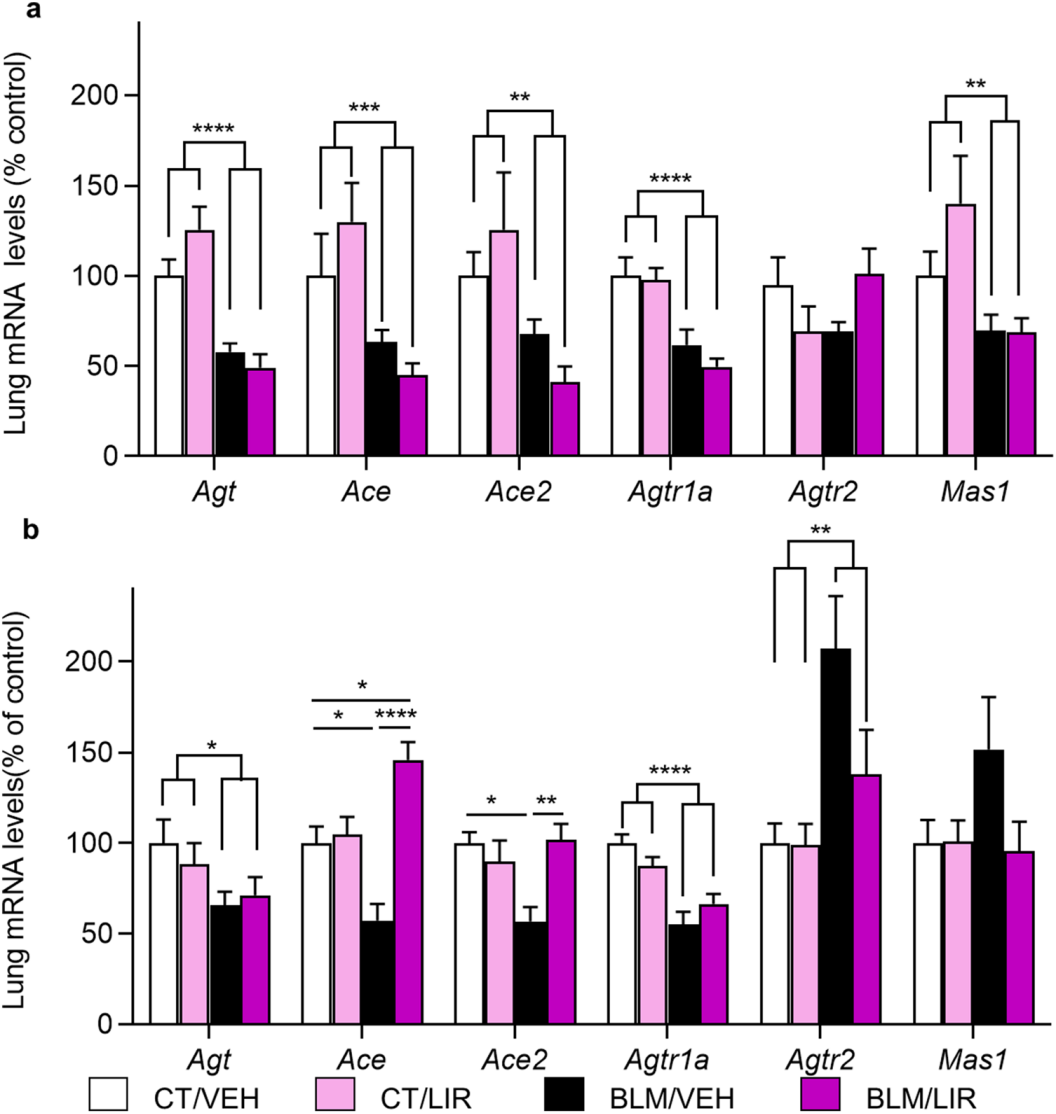
mRNA expression of Renin-Angiotensin System (RAS) components in the lung, normalized to CT/VEH group. Bars represent mean, and error bars SEM. Two-way ANOVA following Bonferroni’s multiple comparison test unless otherwise specified. *p ≤ 0.05, **p ≤ 0.01, ***p ≤ 0.001, ****p ≤ 0.0001. (**a**) Day 7 mRNA levels of angiotensinogen (*Agt*), angiotensin I converting enzyme (*Ace*), angiotensin I converting enzyme 2 (*Ace2*), angiotensin II receptor, type 1A (*Agtr1a*), angiotensin II receptor, type 2 (*Agtr2*) and MAS1 proto-oncogene, G protein-coupled receptor (*Mas1*). n = 6–8 per group. Significance represents two-way ANOVA result. (**b**) Day 21 mRNA levels of *Agt*, *Ace*, *Ace2*, *Agtr1a*, *Agtr2* and *Mas1*. n = 6–8 per group. In *Agt*, *Agtr1a* and *Agtr2* significance represents two-way ANOVA result.

On day 7, in BLM-instilled rats it was decreased the mRNA expression of angiotensinogen (*Agt*, *p* ≤ 0.0001), angiotensin I converting enzyme (*Ace*, *p* = 0.0004), angiotensin I converting enzyme 2 (*Ace2*, *p* = 0.0015*)* angiotensin II receptor, type 1A (*Agtr1a*, *p* ≤ 0.0001) and angiotensin 1–7 receptor, MAS1 G protein-coupled receptor (*Mas1*, *p* = 0.0017). Liraglutide did not change the expression levels of any of the RAS components at 7D time point (Fig. 6a). In day 21, there was also a lower mRNA expression of the two enzymes vs. CT/VEH group (*Ace* 1.43-fold decrease, *p* = 0.0288; *Ace2* 1.43-fold decrease, *p* = 0.0136, Fig. 6b). LIR treatment in BLM-animals increased the mRNA levels of these enzymes (*Ace*: 2.55-fold increase vs. BLM/VEH, *p* ≤ 0.0001; *Ace2*: 1.79-fold increase vs. BLM/VEH, *p* = 0.0070, Fig. 6b), reaching similar levels than in CT/VEH group. Bleomycin instillation reduces *Agt* and *Agtr1a* mRNA expression (*Agt,* *p* = 0.044; *Agtr1a,* *p* ≤ 0.0001) in BLM- rats and LIR treatment did not change it (Fig. 6b). In contrast, Angiotensin II receptor, type 2 (*Agtr2*) mRNA expression was markedly increased in day 21 after bleomycin instillation (2.07-fold increase, *p* = 0.0044), and LIR treatment substantially reduces its expression (*p* = 0.0628, NS, Fig. 6b). Angiotensin 1–7 receptor, MAS1, mRNA expression was not significantly modified (Fig. 6b), reflecting that LIR regulates the expression of the different components of RAS, modulating the degree of activation of the two RAS branches in the lung.

### Liraglutide treatment prevents day 21 right ventricle hypertrophy observed in bleomycin-instilled rats

**Figure 7:**
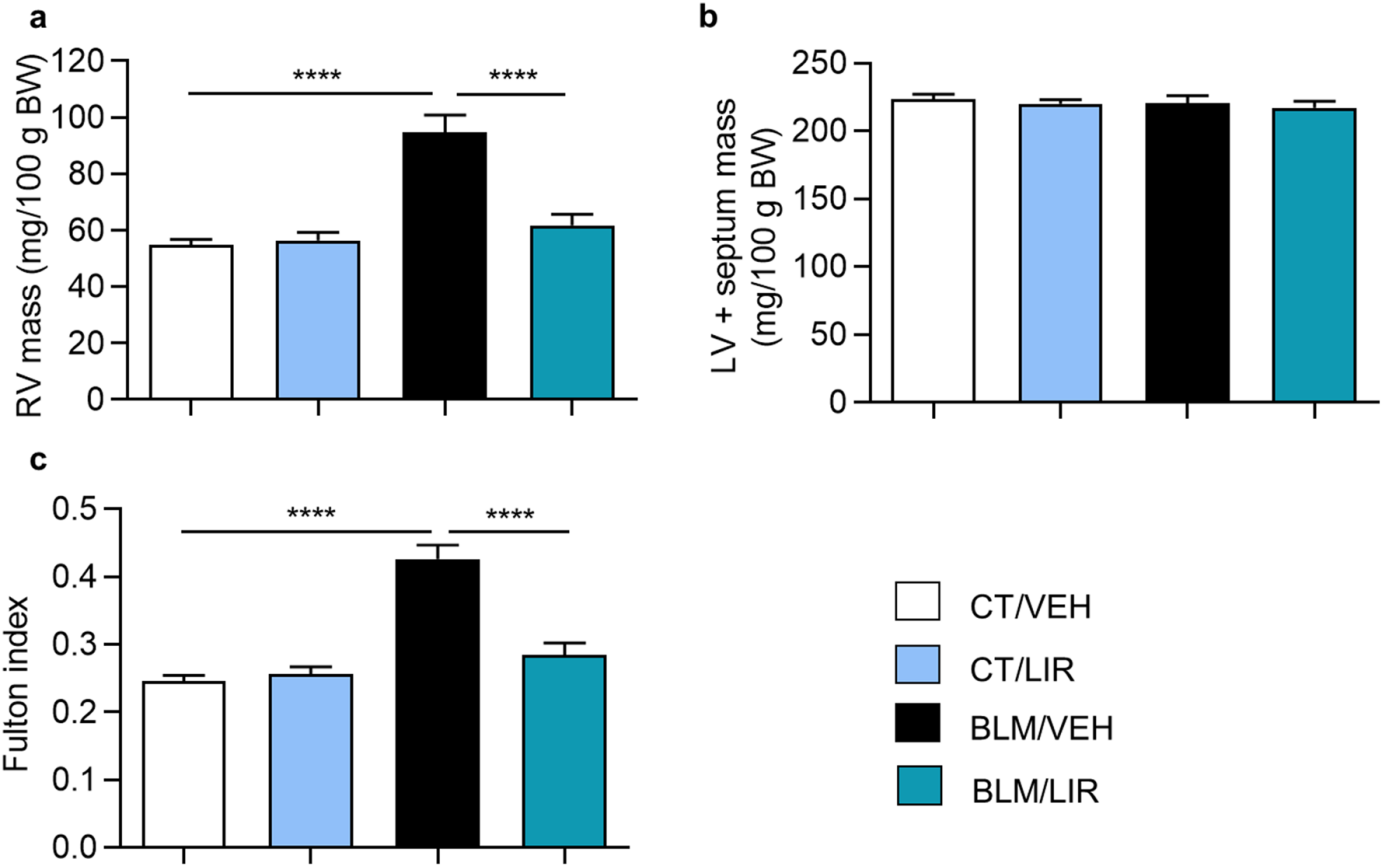
Day 21 heart ventricular masses. Bars represent mean, and error bars SEM. n = 8 in CT groups and n = 12 in BLM groups. Two-way ANOVA following Bonferroni’s multiple comparison test. ****p < 0.0001. (**a**) Right Ventricle (RV) mass normalized to 100 g of body weight (BW). (**b**) Left ventricle plus septum (LV + S) mass, normalized to 100 g of body weight (**c**) Fulton index, a marker of ventricular hypertrophy. Values represent the product of RV weight divided by LV + S weight.

Bleomycin-treated rats, showed increased right ventricle (RV) mass compared to VEH-treated group (Fig. 7a, 94.65 ± 6.19 vs. 54.92 ± 1.89 mg/100 g BW, *p* ≤ 0.0001), whereas no differences were observed in left ventricle plus septum (LV + S) mass (Fig. 7b), at day 21. The Fulton’s Index was increased in BLM-instilled animals too (Fig. 7c, 0.43 ± 0.02 vs. 0.25 ± 0.01, *p* ≤ 0 0.0001). LIR administration to BLM-treated rats completely restored RV mass (Fig. 7a, 61.8 ± 3.89 mg/100 g BW, *p* ≤ 0.0001), and as expected, no differences were observed again in LV + S mass (Fig. 7b). Since LIR reduced the Fulton´s Index (Fig. 7c, 0.29 ± 0.02, *p* ≤ 0.0001) in BLM-treated rats.

### Effect of liraglutide treatment during fibrotic phase in extracellular matrix components

**Figure 8:**
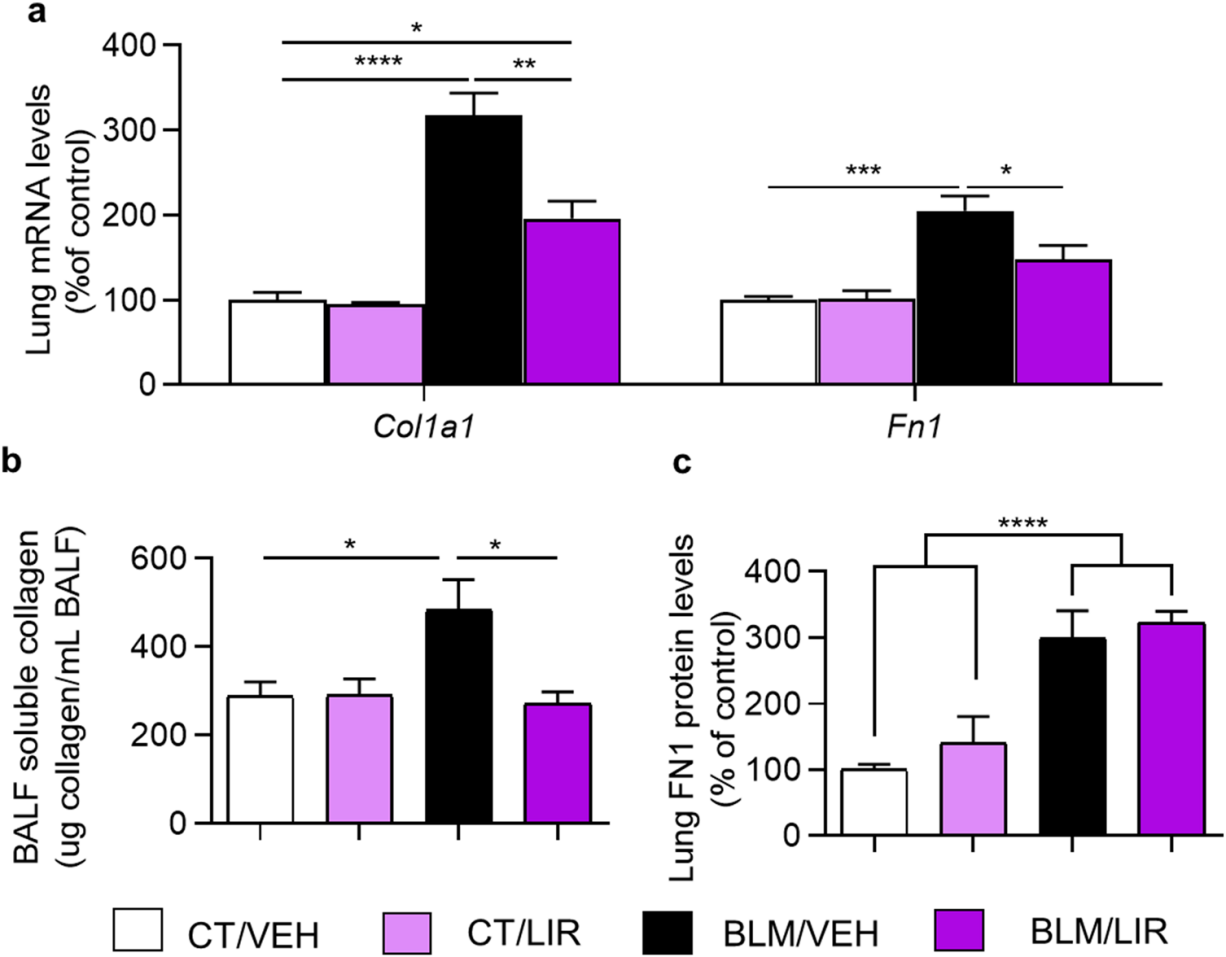
Effect of liraglutide treatment during fibrotic phase over extracellular matrix elements. Bars represent mean, and error bars SEM. Two-way ANOVA following Bonferroni’s multiple comparison test unless otherwise specified. *p ≤ 0.05, **p ≤ 0.01, ***p ≤ 0.001, ****p ≤ 0.0001. (**a**) Day 21 mRNA levels of collagen type I alpha 1 chain (*Col1a1*) and fibronectin-1 (*Fn1*) normalized to CT/VEH group. n = 5–7 per group. (**b**) Day 21 total bronchoalveolar lavage (BAL) soluble collagen. Values represent µg of soluble collagen per mL of BAL. n = 3–7 per group. (**c**) Day 21 protein levels of fibronectin1 (FN1) normalized to CT/VEH group. n = 5–7 per group. Significance represents two-way ANOVA result.

As we previously have shown, bleomycin instillation statistically increases the mRNA expression level of *col1a1* and *fn1 *(Fig. 8a). Liraglutide treatment once fibrotic process is established, from day 10 to day 20, normalize mRNA expression of these two extracellular matrix elements (*Col1a1* 1.62-fold decrease vs. BLM/VEH, *p* = 0.0011; *fn1* 1.39-fold decrease vs. BLM/VEH, *p* = 0.0434; Fig. 8a). Bleomycin instillation also increases BAL soluble collagen levels (482.38 ± 69.79 µg/mL vs. 287.52 ± 33.12 µg/mL; *p* = 0.033; Fig. 8b) and pulmonary tissue FN1 protein levels (2.98-fold increase vs. CT/VEH; *p* = 0.0006; Fig. 8c). Liraglutide completely normalizes BAL soluble collagen protein levels (271.49 ± 26.74 µg/mL vs. 482.38 ± 69.79 µg/mL; *p* = 0.0245; Fig. 8b), but not FN1 protein levels (Fig. 8c).

### Effect of bleomycin instillation and liraglutide treatment over GLP-1R

GLP-1 receptor mRNA and protein were detected in lung tissue (Supplementary Fig. S2). Bleomycin instillation decreases day 7 and day 21 *Glp1r* mRNA expression (day 7 *p* = 0.0022; day 21 *p* ≤ 0.0001; Supplementary Fig. S2a); and liraglutide treatment restores *Glp1r* mRNA expression at both time points (day 7 *p* = 0.0051; day 21 *p* = 0.0035; Supplementary Fig. S2a). We also detect GLP-1 receptor protein on day 7 (Supplementary Fig. S2b) and day 21 (Supplementary Fig. S2c).

## Discussion

Interstitial Pulmonary Fibrosis is a deleterious and progressive disease of the lung with fatal course and not well-understood pathophysiology. Although the incidence of IPF is modest, its rate is increasing in occidental countries, and about 80,000 in the U.S.A. and more than 300,000 patients in the E.U. are suffering this agonic illness. Recent data on Sars-Cov-2 pneumonia suggests a fibrotic sequel in some of the more severe cases^20^, what may increase the incidence in next years. IPF survival ranges 3.8 years from diagnostics, being shorter than many of common neoplasia. Up to date, there are not efficient treatments to fight this disease; despite few drugs have some palliative effects, as nintedanib and pirfenidone^5^. Therefore, the search of new therapeutic options for IPF is mandatory. Our previous and other authors´ work showed that GLP-1 and the agonists of the GLP-1R, highly expressed in pulmonary tissues, promote very potent effects in the lungs, affecting production and secretion of surfactant lipids^11,12^ and proteins^13^, regulation of the components of the pulmonary RAS and vasoconstriction/vasodilatation balance^14,15^; and inflammatory mediators^16^. Since it becomes a challenging opportunity to test the GLP-1 receptor agonist, liraglutide, in an experimental model of IPF and if it has any potential therapeutic capacities in this dramatic disease.

In the present work, we studied the effect of liraglutide administration during the inflammatory phase of the well-established animal model of IPF induced by intratracheal instillation of BLM, and its consequences at two different moments: day 7 in the middle of the inflammatory phase induced by BLM instillation, and day 21 at beginning of the pro-fibrotic phase that develops after BLM-induced injury. In addition, we have studied the effect of liraglutide administration after finished the inflammatory phase and once the fibrosis process initiated upon fibrosis markers (Fig. 8).This model has been shown to have several advantages, highlighting that it resembles histological and functional changes of human lung fibrosis and fulfils the criteria for a good animal model of IPF^6^, is quite easy to perform, and widely accessible and reproducible. This model, reproduces two phases of the disease: an initial inflammatory phase ranging 8–10 days, and a subsequent second phase where collagen interstitial depot predominates, lasting up to 25–27 days after BLM instillation^7^. This model has been widely used to study experimental IPF and current palliative drugs were tested in it.

It has been previously described that the fibrotic progression is promoted by different cytokines. TGF-β1 is the profibrotic cytokine considered the most important molecule to stimulate extracellular matrix synthesis^21^. CTGF is also an extracellular matrix cytokine involved in profibrotic action of TGF-β1^22^. These two cytokines together drive the myofibroblast differentiation, the starring cell type involved in synthesis and secretion of extracellular matrix in fibrotic responses^4^. The presence of myofibroblast in the tissue might be revealed by identifying specific local markers. ACTA2, is the most specific, reliable, and used marker for the identification and quantification of myofibroblast during lung fibrosis^23^. BLM administration induced a differential expression of those cytokines and myofibroblast marker *Acta2*. Thus, in the initial inflammatory phase just *Ctgf* mRNA expression is increased without modifying *Tgfb1* and *Acta2* mRNA expression. In such phase, the inflammatory events predominate but *Ctgf* elevation reveals the start of underlying mechanisms which promote fibrosis. During active fibrotic phase, in day 21, the expression of these two profibrotic cytokines *Ctgf* and *Tgfb1* and the myofibroblast marker *Acta2* are expected to be markedly elevated, showing a complete pro-fibrotic profile. In our study, liraglutide treatment reverts the elevation of these three molecules, both pro-fibrotic cytokines and the myofibroblast marker *Acta2*. Our results confirmed the initial hypothesis that the GLP-1R activation has positive effects in fibrosis-promoting factors. Previously, it was shown that GLP-1R activation can decrease in a dose-dependent manner, the mRNA expression of *Ctgf* in primary culture of human mesangial cells^24^, which agrees with our results.

However, what is relevant and determines the course of the fibrotic process is the collagen depot in the interstitial space of lungs. This is part of the reparative response of the tissues to severe injury accompanied by inflammation. In many tissues and conditions, this reparative response is just partially regenerative substituting some of the original cells by collagen fibres. Collagen type I alpha 1 chain (COL1A1) is the best representative monomer of collagen type I and the most abundant in the ECM^25,26^. Different markers may reveal the presence of excessive collagen depot. OH-Pro is the hydroxylated form of proline, an exclusive component of collagen protein. OH-Pro quantification gives an estimation of total collagen content in a tissue^27^.

We have shown that *Col1a1* expression levels and soluble collagen in BAL are increased from the beginning of the process including both the inflammatory and fibrotic phase in this model. Meanwhile OH-Pro tissue levels were just elevated in the fibrotic phase, revealing an accelerated production rate of collagen at that time. These findings indicate that the transcriptional mechanisms involved in collagen synthesis were activated previously to the beginning of the fibrotic phase. An increase in BAL soluble collagen levels already detected in the inflammatory phase is likely due to alveolar epithelial cells necrosis^28^ before collagen depot. Liragutide treatment was able to block the synthesis and deposition of collagen in the fibrotic phase. Similarly, in other models of cardiac and renal fibrosis, the activation of GLP-1R was able to reduce the fibrotic process^18,29^. For first time, we have described this effect in a lung fibrosis model.

The enzyme prolyl 4-hydroxylase (P4H) plays a critical role to hydroxylate proline residues to 4-Hydroxyproline, that is essential for the stability of the collagen quaternary structure^30^. We have shown that the *P4ha3* mRNA expression levels are increased in the two phases of the BLM model. There is a positive correlation between an increased collagen synthesis and P4H activity in animal models of pulmonary fibrosis and in other human diseases with a fibrotic process^31^. Therapeutic inhibition of this enzyme is today regarded as a promising strategy to prevent fibrosis. Some P4H inhibitors have been tested and they appear to reduce collagen deposition^32^. Up to date, these inhibitors are not clinically relevant due to their low potency, secondary toxicity and poor selectivity^30^. We here describe for first time that GLP-1R activation can inhibit *P4ha3* markedly at transcriptional level and since it may constitute a new and promising therapeutic option for the treatment of fibrotic diseases.

In addition, we studied the local production of proline (Pro), an essential amino acid of collagen. It constitutes, together with his derivative OH-Pro, at least about 25% of the amino acid chain of collagen fibres^33^. In the healing reparative process of wounds, the local Pro levels become increase by 30–50% due to local Pro biosynthesis^34^. The main biosynthetic route involves the urea cycle. Supraphysiological supplementation with l-Arginine has been shown to increase collagen deposition in wounds^35^. There are three key enzymes involved in the local production of Pro: Arginase 1 (ARG1), ornithine aminotransferase (OAT) and pyrroline-5-carboxylate reductase (PYCR); of which we have studied the first and the last of this synthetic route. Bleomycin administration increases mRNA expression level of *Arg1* only in fibrotic phase, but its enzymatic activity is increased in the two phases, what corresponds to the previously reported by Endo et al.^36^. For first time, we described that GLP-1R activation lowers this enzyme at the transcriptional and activity levels. On the other hand, PYCR has three different isoforms: type 1, 2 and 3. Only the *Pycr1* isoform mRNA expression increased after bleomycin administration in the two phases and the others remained unchanged (data not shown). This suggest that PYCR1 is the critical isoform in Pro biosynthesis during pulmonary fibrosis. Liraglutide completely reverts the increase in the mRNA expression of *Pycr1* in the fibrotic phase. Liraglutide was also able to modify the expression of the enzymes of Pro biosynthesis at transcriptional level, in a moment of the fibrotic process when its demand was elevated.

In addition, fibronectin (*Fn1*) contributes to the stability of the collagen fibre network by stablishing crossing-bridges between them, but it is involved in migration and cell adhesion to the ECM^37^, too. We observed that in BLM-model, *Fn1* mRNA expression was increased in both phases studied, and during fibrotic phase (21D) liraglutide reversed this effect. This result suggests that LIR prevents not just the excessive depot of collagen in the tissue, but also the stabilization of the fibres in the ECM.

Complementary and very relevant the tissue structural changes reveal the pathophysiology of the fibrotic process. A specific collagen stain provides valuable information about the fibrosis development and allows an efficient evaluation of the tissue responses^38^. The BLM-model shows histopathological alterations that closely resemble human IPF^39^. Liraglutide was able to reverse at least partially the fibrosis, even at the structural level. It was observed a reduction in collagen fibres deposit, what corresponds with the reduction described in OH-Pro levels and *Col1a1* expression. Liraglutide produces a remarkable structural improvement in pulmonary tissue in this model, which correlates with a functional amelioration also reflected by the increase in body weight observed in LIR-treated rats. Bleomycin administration decreases body weight gain due to alveolar epithelial cells necrosis and alveolitis^40^, and since reduced pulmonary function. Liraglutide administration was solidly proven to reduce food intake^41^, and this effect can affect negatively in the body weight of animals. However, we found an increment in the body weight gain in those ill rats by BLM-instillation, induced by LIR administration, which indicates a functional amelioration in the physiopathological process and a reparative response of the tissue.

Lung surfactant system is critical to maintain alveoli open and functional. Surfactant proteins (SFTPs) plays a fundamental role in lung surfactant stability. Bleomycin reduces mRNA expression of all SFTPs studied in inflammatory and fibrotic phase, as previously shown in this animal model^42^. Liraglutide administration during the inflammatory phase restores SFTPs mRNA expression to the level of control group in fibrotic phase. The capacity of liraglutide to stimulate the secretion and the production of lung surfactant improves alveolar stability and gas exchange, which contributes to a better respiratory function. Previously, we have shown that GLP-1 receptor agonists are able to restore the normal levels of mRNA of these proteins in other lung pathological states, such as foetal lung hypoplasia^13^ and type 1 diabetes^14^.

IPF is accompanied by pulmonary arterial hypertension (PAH), which is an important predicting fatal prognosis factor in humans^43^. PAH affects right cardiac ventricle promoting hypertrophy, which itself is a major marker of functional altered status and prognosis in PAH, and consequently in IPF^44^. In the BLM-IPF model excessive fibres accumulation disrupts interalveolar interstitium and increase resistance to blood flow in the pulmonary vascular bed promoting PAH^45^ and subsequent right ventricle hypertrophy. Liraglutide treatment prevented the development of right ventricle hypertrophy, as we and others described previously in models of lung pathology^14,17^.

In this context, the Renin Angiotensin System (RAS) is crucial in the regulation of cardiovascular system, and it has a special representation in the pulmonary vascular bed playing a fundamental role in the regulation of vessels´ tone. RAS is a complex set of molecules, enzymes, and receptors, organized in two different branches with eventual antagonist functions. Angiotensinogen is the precursor peptide of the RAS active molecules and expressed locally in lung tissue^46^. BLM markedly reduces *Agt* mRNA expression levels in inflammatory phase (day 7). These changes reveal different vascular events occurring in each time phase of the BLM model: in the first inflammatory phase, the cytokines produced by the affected tissue induce vasodilatation; meanwhile in the last pro-fibrotic phase the interstitial depot of collagen favours increased vascular resistance.

Bleomycin instillation decreases *Ace* (Angiotensin-Converting-Enzime) and *Ace2* mRNA expression, and this decrease is even more marked on day 21. Previously, other studies had demonstrated that these enzymes are downregulated in animal models of IPF^47,48^, hypothetically due to the endothelial/epithelial injury. LIR treatment additionally decreases the expression of these two enzymes on day 7, but LIR remarkably reverted the expression levels of *Ace* and *Ace2* on day 21. Previous studies of our group in different animal models, showed that LIR restores *Ace* and *Ace2* mRNA expression levels in type 1 diabetes animals, and it consistently induced an overexpression of these two enzymes when administered to normal controls^14^.

Moreover, the angiotensin II receptor, type 1A (*Agtr1a*) mRNA expression decrease in the two phases of the BLM model, what agrees with the results of immunohistochemistry studies carried out in FPI patients^49^. In contrast, angiotensin II receptor, type 2 (*Agtr2*) mRNA expression was markedly increased during fibrotic phase, in agreement with other studies^49–51^. Liraglutide treatment also prevented this effect. Interestingly, GLP-1R agonists modulate AGTR2 in other tissues as shown in a model of cardiac fibrosis, where the expression of AGTR2 is reduced and GLP-1R activation increases its expression^19^. Even further, MAS1 is the receptor for Ang (1–7), the product of AngII cleavage by ACE2, and it plays a very important role in the vasodilatory, anti-apoptotic and anti-proliferative effects of Ang (1–7)^52^. We have previously reported that ACE2/Ang (1–7)/MAS1 axis was activated and enhanced in lung tissue of weaned pups after GLP-1R activation in an animal model of intrauterine growth restriction^15^. This branch of the pulmonary RAS axis is critical to understand the balance of vascular tone in the lung vascular bed. It fact, the increased activation after liraglutide treatment likely underlying the anti-hypertensive effects of GLP-1R agonists in pulmonary circulation and explains the reversion of the right ventricle hypertrophy we found in the BLM-model, as we previously describe in other models too^14,15^.

As a whole our results show, by the first time, that the administration of liraglutide in the precocious initial phase of lung fibrosis, reduces collagen interstitial deposition and production of precursor materials as proline and hydroxyproline, reduces presence of myofibroblasts in the tissues and the expression of pro-fibrotic cytokines as *Tgfb1* and *Ctgf*. Moreover, liraglutide modulates de activity of the pulmonary angiotensins and their receptors improving lung vascular conditions and preventing right ventricle hypertrophy. In addition, liraglutide increase the expression and protein levels of key surfactant proteins, and all together improve the alveolar histological structure, finally ameliorating clinical condition of the animals. Since liraglutide arises as a new promising molecule with potential therapeutic applicability in lung fibrosis, awaiting to reproduce its effects in clinical studies.

## Methods

### Animals

All experimental procedures were performed in young adult (12 weeks) male Sprague–Dawley rats (200 ± 20 g), obtained from Animal Housing Facility of University of Santiago de Compostela (Santiago de Compostela, Spain). The animals were maintained in the Bio-experimentation Service of the University of Vigo (SB-UVI), on a 12/12 light cycle, at controlled room temperature (22 ± 2 °C) and humidity (50 ± 5%); with ad libitum access to water and standard food (A04; Panlab, Barcelona, Spain). Animals were randomly assigned for each experimental group.

### Drugs

Bleomycin sulphate (BLM) was obtained from Mylan Pharmaceuticals S.L. (Barcelona, Spain); liraglutide ([Lys (γ-Glu-palmitoyl)^26,Arg34^]-GLP-1 [7–37]) was purchased from Bachem (Bubendorf, Switzerland).

### Experimental procedure

Rats were treated with liraglutide (LIR, 100 µg/kg per 12 h dissolved on 0.4% acetic acid, subcutaneously) or vehicle (VEH, 0.9% NaCl solution plus 0.4% acetic acid). They were treated using two different patterns. In the first pattern, animals were treated between day-1 and day 6 after endotracheal instillation of BLM. In the second pattern, they were treated between day 10 and day 20. On day 0, rats were intra-tracheal injected with BLM (2.5 mg/kg body weight, dissolved in 200 µL of sterile 0.9% NaCl) or 200 µL of 0.9% NaCl (CT), as previously described^53^. To ensure correct BLM administration, we used only the animals that showed a sustained weight loss at least until day 4. This model characteristically develops in two consecutive phases: a first phase predominantly inflammatory from day 1 to about day 9, and a second phase predominantly showing deposit of collagen in the lung interstitial from day 9 onwards.

Groups of eight rats were killed on day 7 and 21 after BLM or NaCl instillation by an overdose of sodium pentobarbital (200 mg/kg, intraperitoneally, Sigma-Aldrich). Trunk blood was obtained directly from abdominal artery trough a puncture trough exposed artery. Blood was collected in a BD Vacutainer tube containing K3EDTA (15%) and aprotinin (250 KIU) (Beckton Dickinson, catalog number 361017), and stored in ice prior to further processing. Animal death was confirmed by abdominal artery exsanguination. Caudal lobe of right lung was removed and stored at − 80 °C for RNA and protein extraction. The medial lobe of right lung was also removed and stored at − 80 °C for OH-Pro quantification and arginase activity assay. Left lung was weighted and then immediately cannulated by the principal bronchia with a 22-gauge plastic cannula (Braun, Melsungen, Germany) and rinsed with 2 mL of cold sterile 0.9% NaCl solution to collect BAL. BAL was stored on ice prior to further processing.

The heart was excised, left and right atrium removed, and RV was dissected from LV + S and weighted separately. Fulton´s Index was calculated as the ratio of RV mass/LV + S mass, and it was used as marker of RV hypertrophy.

### Broncho-alveolar lavage and blood processing

Blood tubes were centrifuged at 2000 g for 10 min at 4 °C. Supernatant was aliquoted and stored at − 20 °C for further analysis.

500 µL of the BAL collected from the animals was centrifuged at 300 g for 10 min at 4 °C. Supernatant was aliquoted and stored at − 20 °C for further analysis.

### Analysis of soluble collagen

Total BAL soluble lung collagen content was quantified using Sirius Red stain absorbance method as previously described^54^. 100 µL of 6 standard-curve samples of Collagen from calf skin, ranging a concentration between 50–1.56 mg/dL (Sigma cat. C9791) and 100 µL of each sample were loaded in duplicate in a 96-well flat bottom plate. Then, 150 µL of collagen-binding dye (0.1% Sirius red stain [Direct red 80, Sigma-Aldrich, cat. 365548] in saturated picric acid solution) was loaded in each well and plate was incubated 60 min at 37 °C. The plate was centrifuged at 3000 g for 10 min, supernatant was discharged, pellet was washed by absolute ethanol for 2 min, and plate was centrifuged at 3000 g for 10 min. Supernatant was discarded and collagen-dye pellet was resuspended using 200 µL of 0.5 M NaOH and incubated at 37 °C for 30 min in obscurity. Finally, 96-well plate dissolved collagen dye pellets were measured spectrophotometrically at 540 nm using a plate reader EnVision (Perkin Elmer, MA, USA). Results were represented as µg of collagen per mL of BAL.

### Quantification of TGF-β1 levels by ELISA

Plasma and BAL TGF-B1 levels were quantified by ELISA using a commercial kit (Human TGF beta-1 DuoSet ELISA, cat. DY240-05; and DuoSet ELISA Ancillary Reagent Kit 1, cat. DY007. R&D systems, MN, USA). 175 µL of plasma per sample was activated by acidification with 35 µL of 1 N HCl. Samples were incubated 10 min at room temperature and them they were neutralised using 35 µL of 1.2 N NaOH/ 0.5 M HEPES. 40 µL of plasma per sample was activated by acidification with 20 µL of 1 N HCl. Samples were incubated 10 min at room temperature and them they were neutralised using 20 µL of 1.2 N NaOH/ 0.5 M HEPES. Activated BAL Samples were used undiluted. Activated plasma samples were diluted to a final 20-fold dilution using reagent diluent provided by the kit. 100 µL of each standard and sample were loaded in a previously precoated 96-well plate following manufacturer´s instructions and the rest of the protocol has advanced following them. Results were represented as pg/mL for BAL samples and as ng/mL for plasma samples.

### Total RNA extraction and synthesis of cDNA

RNA from right lung caudal lobe was isolated by the single step acid guanidinium thiocyanate–phenol–chloroform extraction^55^. RNA was quantified spectrophotometrically using Nanodrop 2000c (Thermo Scientific, Delaware, United States) and purity of RNA was determined by the ratios of absorbance (260/280 ≥ 2.0) and [260/230 (2.0–2.2)]. Quality of RNA was determined by running 2 µg of RNA in a non-denaturing agarose gel electrophoresis and check the integrity of rRNA 28S and 18S bands. Only samples with a good purity and quality were used for reverse transcription (RT) reaction.

The RT was performed on 2 µg of total RNA in a reaction containing: 100 pmol Random Hexamer primers, 1X RevertAid RT Buffer, dNTP Mix 10 mM each, 1 U/µL Ribolock RNase inhibitor, and 10 U/µ RevertAid Reverse Transcriptase (Thermo Scientific, Massachusetts, United States). The reaction was carried out in two steps. Firstly, RNA was incubated with Random Primers 65 °C for 5 min. Secondly, all the components were incubated 10 min at 25 °C, 60 min at 42 °C and 10 min at 70 °C. Final reaction volume was 20 µL.

### mRNA expression levels by real-time PCR

Semi-quantitative real time PCR reactions were performed using an apparatus Agilent Technologies 7900HT (Applied Biosystems, CA, United States). mRNA expression of *Ace*, *Ace2* and the housekeeping *18S* was carried out with predesigned Real Time Ready Single assay (*Ace*: ID503125, *Ace2*: ID505454 and *18S*: ID502300) and Fast Start Universal Probe Master (Roche Diagnostic, Indianapolis, United States) on 10 ng cDNA. mRNa expression of other genes was carried out using PowerUp Sybr Green Master Mix (Applied Biosystems, California, United States). Primer pairs were designed using Primer Blast Software^56^. Primers pairs, cDNA dilution used and housekeeping for normalization, are described in Supplementary Table S1. cDNA has been amplified using the following protocol: 2 min at 50 °C, 2 min at 95 °C and 40 cycles of 15 s at 95 °C, 15 s at 57 °C and 1 min at 60 °C. Relative mRNA expression was determined by the 2^−ΔΔCt^ method^57^ using the SDS 2.4.1 Software (Applied Biosystems, Foster City, Ca, United States). Results were represented as a percentage of variation in respect to control (CT/VEH) group.

### Total protein tissue extraction

Total caudal lung proteins were obtained by homogenization in RIPA lysis buffer with a protease inhibitor cocktail (cOmplete™ Protease Inhibitor Cocktail, cat. 4693116001). After homogenization, samples were centrifuged at 12,000 G for 20 min to remove cell debris. Supernatant was aliquoted and stored at − 80 °C for further analysis.

### Protein expression levels using Western-Blot

Lung homogenate´s total protein level was quantified espectrophotometrically using a commercial Bradford assay (cat. 5000006, Bio-Rad Laboratories, München, Germany). 30 µg of proteins were boiled at 95° with loading buffer; and then they were separated in a SDS-PAGE, using 7.5% precast gels (cat. 4561026, Bio-Rad Laboratories, München, Germany). Proteins separated in the gel were transferred to a PVDF membrane (cat. 1704156, Bio-Rad Laboratories, München, Germany) using a TransBlot Turbo apparatus (Bio-Rad Laboratories, München, Germany).

Membranes were blocked using a blocking solution (Tris Buffered Saline (TBS)-0.1% Tween 20 with 5% skimmed milk powder) for 1 h and at room temperature. Membranes were incubated overnight at 4 °C with the primary antibodies diluted in the blocking solution against BACT (1:5000, cat. A5441, Sigma-Aldrich); FN-1 (1:1000, cat. SAB4500974, Sigma-Aldrich) and GLP-1R (1:400, cat. sc-66911 Santa Cruz Biotechnology). Membranes were washed 3 times with TBS-0.1% Tween 20 and incubated1 hour at room temperature with a secondary antibody diluted in blocking solution. For BACT an anti-mouse IgG horseradish peroxidase conjugated (1:3000, cat. 170-6516, Bio-Rad Laboratories, München, Germany) and for FN-1 and GLP-1R an anti-rabbit IgG horseradish peroxidase conjugated (1:10,000, cat. 111-035-144, Jackson ImmunoResearch Europe, Ely, United Kingdom). Finally, membranes were washed 3 times with TBS-0.1% Tween 20 and membranes were incubated with the chemiluminescent substrate (Clarity Western ECL substrate, cat. 1705060, Bio-Rad Laboratories, München, Germany). Luminescence signal was visualized using Bio-Rad ChemiDoc XRS system and, in case of BACT and FN1, quantified with Bio-Rad ChemiDoc XRS Image Lab 3.0 software. The capture time was selected at the best point of the slope for signals (every 10 s), avoiding band saturation and allowing quantifications with a minimal variation. Band intensities are normalized using BACT as housekeeping protein, and data represents as percentage of variation versus CT/VEH group.

### Hydroxyproline quantification

A piece of right lung medial lobe was homogenized in deionized water (10 μL per mg of tissue) using a disperser. 600 μL of tissue homogenate was transferred to a Pirex tube with screw top, and same volume of 12 N HCl was added. Tissue homogenate was digested in an oven for 3 h at 120 °C. Afterwards, 1 mL of digested sample was translated to a glass tube that contains 15 mg of activated charcoal and 1 mL of deionized water. This tube was centrifuged at 400 G for 15 min, and 1 mL of the supernatant was disposed in a new glass tube. Samples were neutralized in presence of 10 μL of 1% phenolphthalein with 290 μL of NaOH 10 N. Hydroxyproline quantification was determined using a previously described method^54^. Results were represented as micrograms of Hydroxyproline per milligram of wet tissue.

### Arginase activity assay

This assay was previously described by Wynn^54^ with minor variations. A piece of lung medial lobe was homogenized in lysis buffer, and activated solution was incubated by arginase substrate solution for 24 h. After that, the urea generated was quantified using a commercial assay (UREA-37, Spinreact, Girona, Spain). Total protein levels were determined in lung homogenate using a commercial Bradford assay (cat. 5000006, Bio-Rad Laboratories, München, Germany). Data were represented as micrograms of urea generated in 24-h period, per mg of protein in lung homogenate.

### Histological analysis

In day 21, after BAL sampling, left lung lobe was excised in 3 mm-thick chunks and fixed by immersion in 10% neutral-buffered formalin for 24 h. After fixation, chunks were included in paraffin and 5 µm slices were sectioned using a microtome. The slices were stained using Masson’s trichrome staining kit (Sigma-Aldrich cat. HT15) following the manufacturer´s instructions. Images were acquired using Nikon NiE microscope. To evaluate the severity of fibrosis 16 micrographs per sample were taken randomly at 200 ×. Images were analysed in double blind study by 3 different technicians. Each micrograph was scored using modified Ashcroft score that is described previously^58^. The average for the scoring for each sample of the 3 different technicians were represented.

### Statistical analysis

Data representation and statistical analysis was performed using Graph Pad Prism 8.0.2 (GraphPad Software, LaJoya, CA, United States). The data were represented as mean ± standard error of the mean (SEM). Comparisons between experimental groups were analysed by two-way ANOVA. In the study was defined two different independent variables: fibrosis and LIR treatment. When the interaction between them where statistically significant a Bonferroni’s multiple comparison test was performed. Statistical differences respect to CT/VEH and BLM/VEH groups were represented or two-way ANOVA result unless indicated. By convention, a value of *p* ≤ 0.05 was accepted as statistically significant.

### Study approval

The animal experiments were developed in accordance with European Community (2010/63/UE) and Spanish laws for animal experimentation (RD53/2013). They were approved by the corresponding authorised organ of the regional government (CEIC, Xunta de Galicia, Spain, reference number: ES360570215601/17/INV.MED.02. OUTROS04/LCGM/02).

## Supplementary information

Supplementary Information

## Data Availability

The datasets generated and analysed during the current study are available from the corresponding author on reasonable request.
