# Supplementary material for: GLP-1 receptor agonist ameliorates experimental lung fibrosis
Source: Sci Rep. 2020 Oct 22;10:18091. doi: 10.1038/s41598-020-74912-1 (PMC7581713; doi:10.1038/s41598-020-74912-1)
Supplement: Supplementary file 1 — Supplementary Information [file 41598_2020_74912_MOESM1_ESM.pdf]

GLP-1 receptor agonist ameliorates experimental lung fibrosis

Juan Fandiño Gómez, Laura Toba Estévez, Lucas González Matías, Yolanda Diz Chaves & Federico Mallo Ferrer*

AUTHORS AFFILIATION:

Laboratory of Endocrinology (LabEndo) – The Biomedical Research Centre (CINBIO) – University of Vigo – Campus Universitario de Vigo (CUVI) – e-36310 – Vigo – SPAIN.

SUPPLEMENTARY INFORMATION

Supplementary Figure S1

a

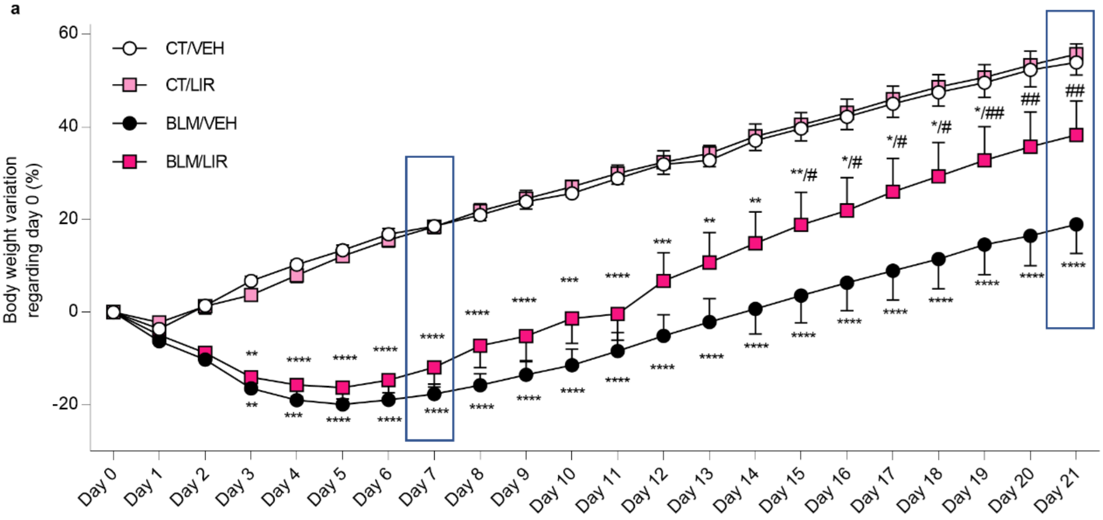

b

Group	Slope Value (m)
CT/VEH	2.47 ±0.22
CT/LIR	2.65 ±0.26
BLM/VEH	2.62 ±0.35
BLM/LIR	3.94 ±0.17 (**/##)

Supplementary Figure S1. Body weight variation during the experimental period. Control (CT) groups n=8; Bleomycin (BLM) groups n=12. **(a)** Body weight variation represented as a percentage of body weight in respect to day 0 of experiment. Two-way ANOVA following Bonferroni´s multiple comparison test. * p≤0.05; ** p≤0.01; *** p≤0.001; **** p≤0.0001 vs. CT/VEH; # p≤0.05; ## p≤0.01 vs. BLM/VEH. **(b)** Calculated slope of the body weight gain between day 7 and day 21 of the experiment; data represented as gram of body weight gain per 100 g per day (g/100g/day). Two-way ANOVA following Bonferroni´s multiple comparison test. ** p≤0.01 vs CT/VEH; ## p≤0.01 vs BLM/VEH.

Supplementary Figure S2

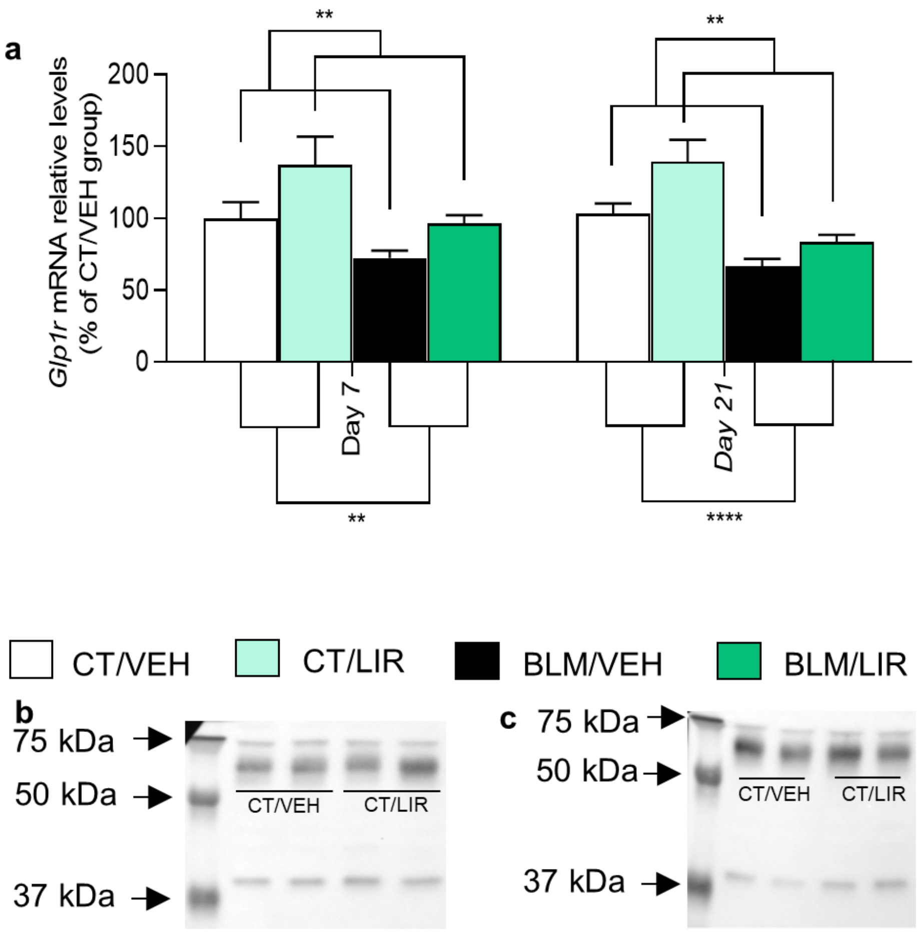

Supplementary Figure S2. mRNA expression of GLP-1 receptor in lung tissue. Bars represent mean, and error bars SEM. Two-way ANOVA test. ** $p \leq 0.01$; **** $p \leq 0.0001$. **(a)** Day 7 and day 21 mRNA levels of glucagon-like peptide 1 receptor (*Glp1r*) normalized to C T/VEH group. n= 5-8 per group. **(b)** Representative Western-Blot cropped membrane of Day 7 GLP-1R protein. **(c)** Representative Western-Blot cropped membrane of Day 21 GLP-1R protein.

Supplementary Table T1

RT PCR primers used.

Gene (accesion number)	Forward primer sequence	Reverse primer sequence	Product length (bp)	PCR efficiency (%)	cDNA dilution	Housekeeping gene
Actb (NM_031144.3)	5´-CACCATGTACCCAGGCATTG-3´	5´-CCTGCTTGCTGATCCACATC-3´	173	99,8	1\32	
Gapdh (NM_017008.4)	5´-AGCCCAGAACATCATCCCTG-3´	5´-GCATGTCAGATCCACAACGG-3´	136	104,7	1\32	
Acta2 (NM_031004.2)	5´-GGAGATGGCGTGACTCACAA-3´	5´-CGCTCAGCAGTAGTCACGAA-3´	152	101,9	1\32	*Actb*
Agt (NM_134432.2)	5´-GCTGGAGCTAAAGGACACACA-3´	5´-GCAGGTGCTCTTGCTGTAGTA-3´	169	101,2	1\8	*Gapdh*
Agtr1a (NM_030985.4)	5´-TTCGTGGCTTGAGTCCTGTT-3´	5´-GGTGATCACTTTCTGGGAGGG-3´	178	96,6	1\32	*Gapdh*
Agtr2 (NM_012494.3)	5´-CCGTGACCAAGTCTTGAAGATG-3´	5´-AGGGAAGCCAGCAAATGATG-3´	65	99,3	1	*Actb*
Arg1 (NM_017134.3)	5´-GCAGAGACCCAGAAGAATGGAAC-3´	5´-CGGAGTGTTGATGTCAGTGTGAGC-3´	144	101,0	1\8	*Gapdh*
Col1a1 (NM_053304.1)	5´-TGCAACATGGAGACAGGTCA-3´	5´-CGCTTCCATACTCGAACTGG-3´	151	106,3	1\32	*Gapdh*
Ctgf (NM_022266.2)	5´-CGCCAACCGCAAGATTG-3´	5´-CGCTTCCATACTCGAACTGG-3´	69	109,8	1\16	*Gapdh*
Fn1 (NM_019143.2)	5´-CAACTTCTGGTCCTCTCCCG-3´	5´-GGACCCCTGAGCATCTTGAG-3´	158	96,1	1\32	*Gapdh*
Glp-1R (NM_012728.1)	5´-AGTAGTGTGCTCCAAGGGCAT-3´	5´-AAGAAAGTGCGTACCCCACCG-3´	190	106,2	1\32	*Gapdh*
Mas1 (NM_012757.2)	5´-CTGGTCAACCTTTGGGAACCT-3´	5´-AAAGGGTTGGCGCTGCTA-3´	72	101,9	1\2	*Gapdh*
Nkx2-1 (NM_013093.1)	5´-CTTACCAGGACACCATGCGG-3´	5´-CTCATATTCATGCCGCTCGC-3´	118	104,0	1\32	*Gapdh*
P4ha3 (NM_198775.1)	5´-TTCCTTGTCTACAGCCCAGAC-3´	5´- TAGCCCCTCATAGGTGTCCC-3´	156	99,7	1\32	*Gapdh*
Pycr1 (NM_001105857.2)	5´-CTGTGGAAGCCTCCTGTATCC-3´	5´- CGGCTTAACATGGCCAGAAGA-3´	152	106,4	1\4	*Gapdh*
Sftpa1 (NM_001270645.1)	5´-CTGCCAGGATTTCCAGCTTAC-3´	5´-TTGACTGACTGCCCATTGGT-3´	155	100,6	1\32	*Gapdh*
Sftpb (NM_138842.1)	5´-CTGTGCCAAGAGTGTGAGGA-3´	5´-CAAGCAGCTTCAAGGGTAGG-3´	124	108,2	1\32	*Gapdh*
Sftpc (NM_017342.2)	5´-TGCTGCCCCGTGCATCTCAA-3´	5´-TTCACTCAGGGCGAGGCGTT-3´	180	94,3	1\32	*Gapdh*
Tgfb1 (NM_021578.2)	5´-GCTGAACCAAGGAGACGGAA-3´	5´-CCTCGACGTTTGGGACTGAT-3´	114	96,9	1\16	*Gapdh*

This table represent gene name and accession number of NCBI, primer pair sequence, the length of the amplicon, the efficiency of the PCR reaction calculated by the slope of the standard curve, the dilution of the cDNA used for the PCR reaction and the housekeeping gene used for the normalization of the gene expression.
